# Supplementary material for: Synthesis of New S-Triazine Bishydrazino and Bishydrazido-Based Polymers and Their Application in Flame-Retardant Polypropylene Composites
Source: Polymers (Basel). 2022 Feb 17;14(4):784. doi: 10.3390/polym14040784 (PMC8876278; doi:10.3390/polym14040784)

Synthesis of new s-triazine bishydrazino and bishydrazido based polymers and their application in flame retardant polypropylene composites

Ali Aldalbahi [1*], Bander S. AlOtaibi [1], Badr M. Thamer[1] and Ayman El-Faham [1]

Department of Chemistry, College of Science, King Saud University, P.O. Box 2455, Riyadh 11451, Saudi Arabia

*Correspondence: aaldalbahi@ksu.edu.sa; Tel.: 00966114677703

Table of content

Method S1 General method for the synthesis of 2a-c

Method S2 General method for the synthesis of 3a-c

Figure S1 1H and 13C NMR for 2a

Figure S2 1H and 13C NMR for 2b

Figure S3 1H and 13C NMR for 2c

Figure S4: DSC full cycle of 5a-c samples

Figure S5: DSC full cycle of 7a-c samples

Method S1

General method for the synthesis of 2,4-Dichloro-6-substituted-s-triazine derivatives 2a-c 2,4,6-Trichloro-1,3,5-triazine (cyanuric chloride, 20 mmol) was dissolved in acetone (100 mL), and then added to an aqueous solution of amine such as (aniline, p-bromoaniline, or p-methoxyaniline 20 mmol,) in acetone-water (100 mL, 1:1) contained NaHCO3 (20 mmol) at 0 ºC. After complete addition, the reaction mixture was stirred for 2h at 0 ºC followed by addition excess of water. Acetone was removed under vacuum and the solid product was collected by filtration and then dried to afford 2,4-dichloro-6-substrituted s-triazine 2a-c in good yields and purities.

Method S2

General method for the synthesis of 2,4-dihydrazino-6-substtituted-1,3,5-Triaizne derivatives 3a-c Hydrazine hydrate (80%) in acetonitrile (20 mL) was added dropwise to a solution of 2a-c (20 mmol) in ethanol (50 mL) at room temperature. The reaction mixture was stirred under refluxed for 3 h. The excess of solvent and hydrazine was removed under reduced pressure and the crude white solid was collected by filtration, washed with cold ethanol, ether, and then dried at room temperature to give the desired bis-hydrazino derivatives with high purity as observed from TLC (methanol-chloroform; 2:8). The products were used directly to the next step without further purification.

Figure S1: 1H and 13C NMR for 2a

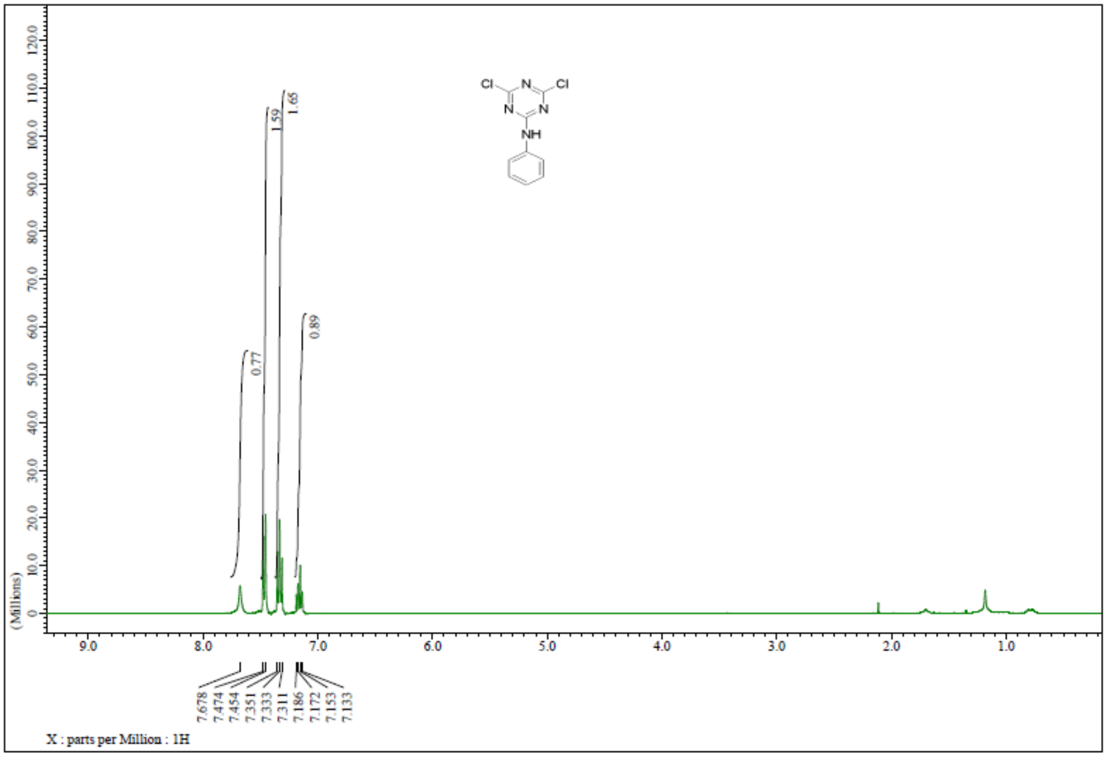

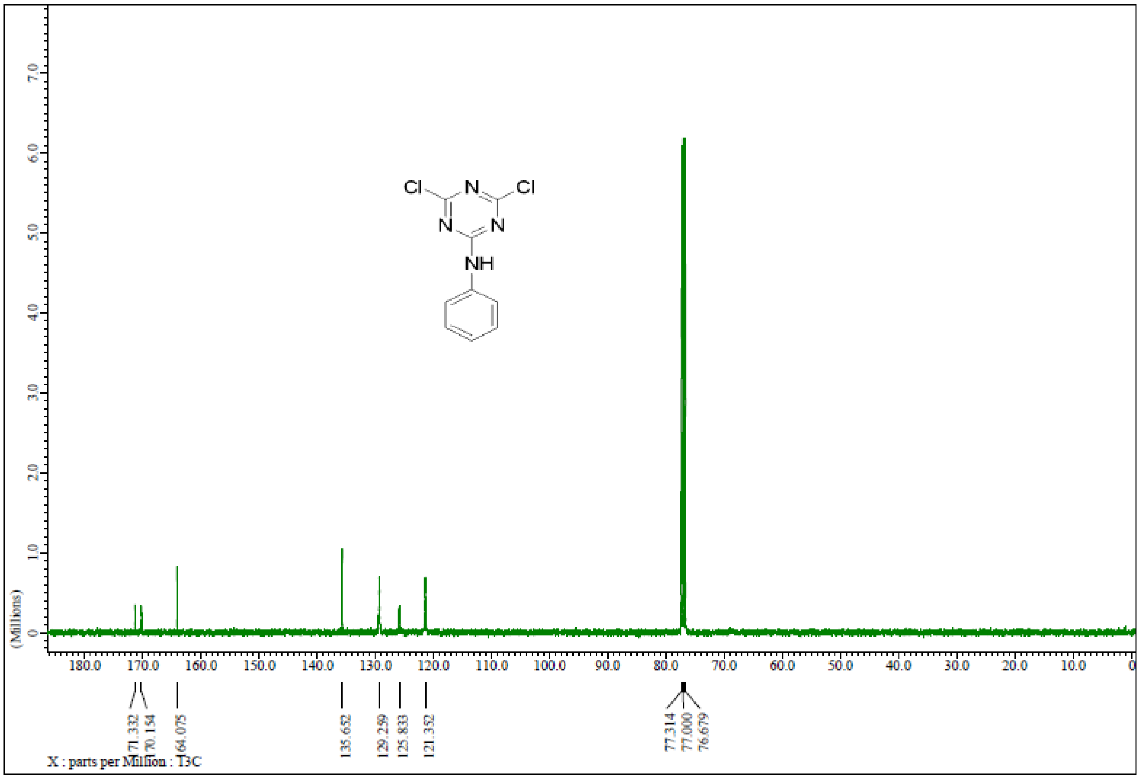

Figure S2: 1H and 13C NMR for 2b

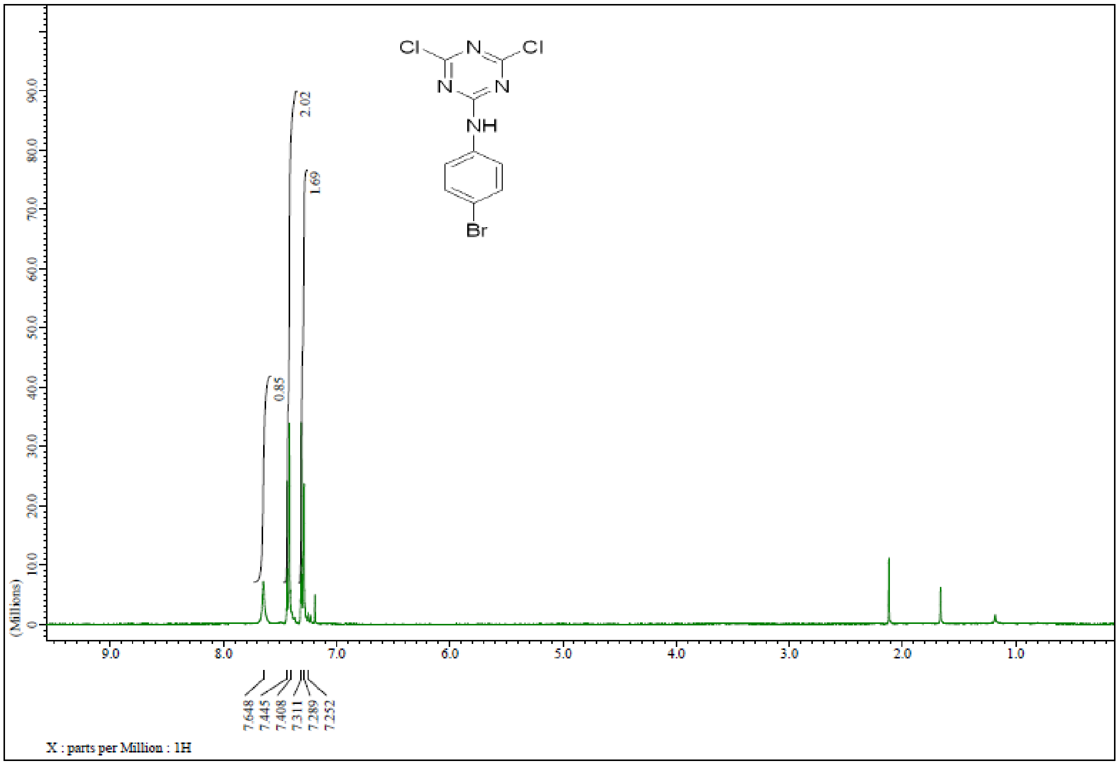

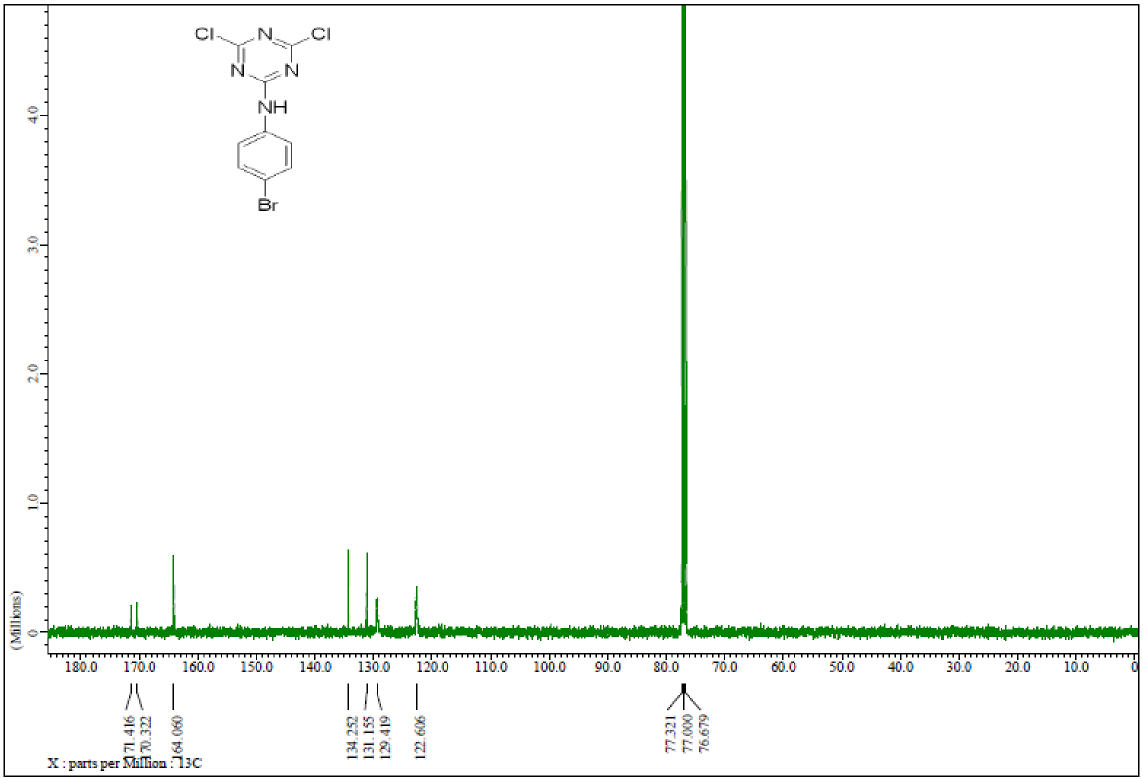

Figure S3: 1H and 13C NMR for 2c

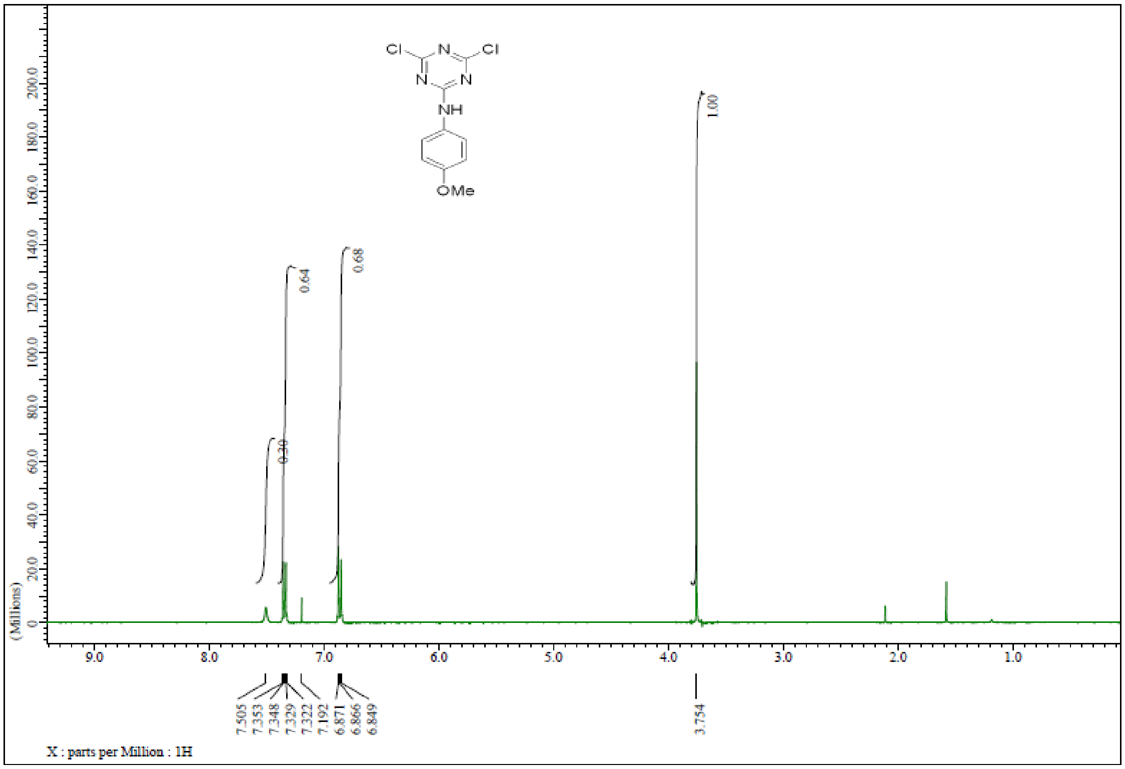

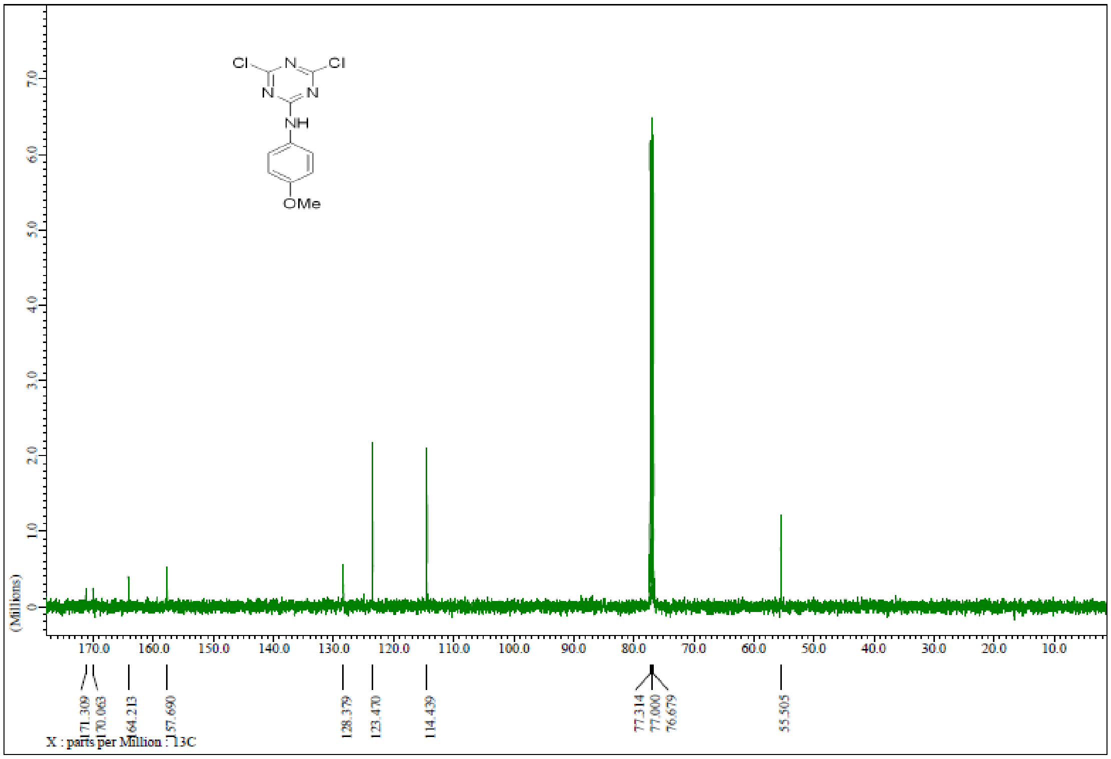

Figure S4: DSC full cycle of 5a-c samples

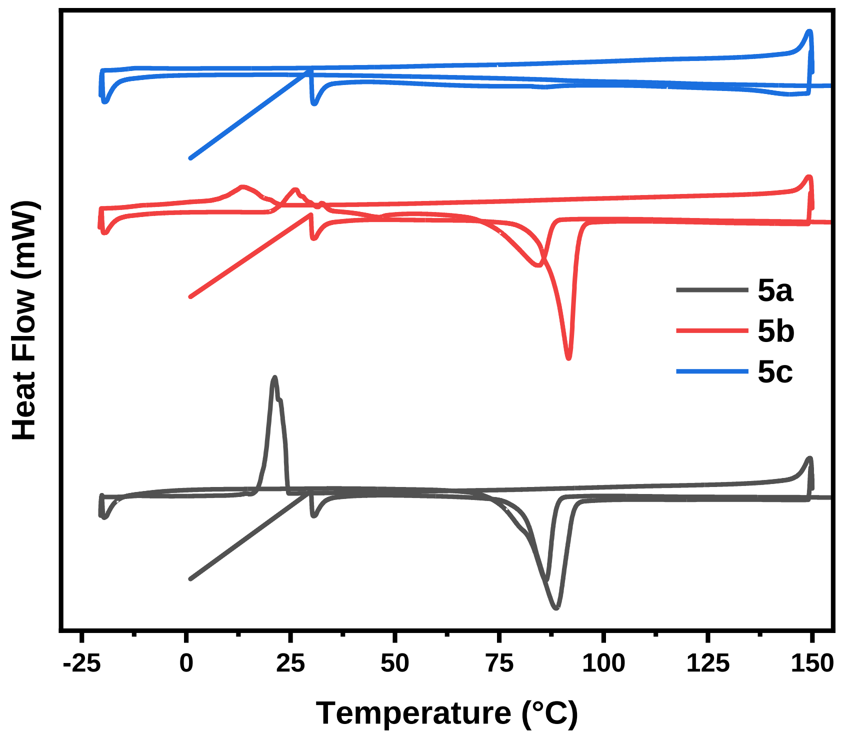

Figure S5: DSC full cycle of 7a-c samples

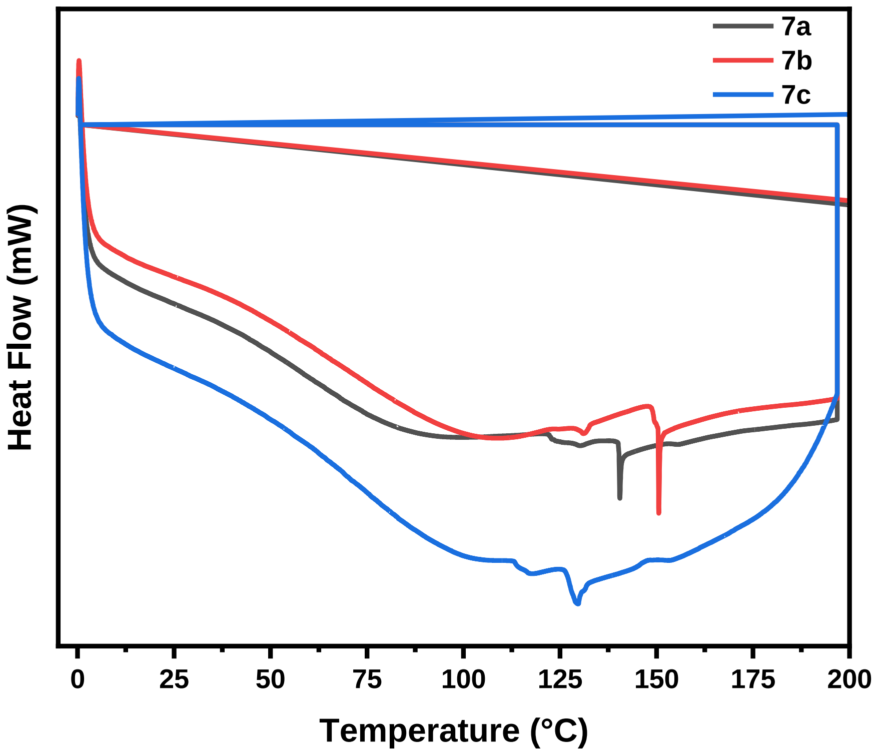

Supplement: Supplementary file 1 [file polymers-14-00784-s001.zip › polymers-1589568-supplementary.pdf]
